# Outcomes in urinary sepsis

**DOI:** 10.1186/cc11982

**Published:** 2013-03-19

**Authors:** C Joya-Montosa, E Trujillo-Garcia, MJ Delgado-Amaya, E Curiel-Balsera

**Affiliations:** 1 Hospital Regional Carlos Haya, Málaga, Spain

## Introduction

Analysis of mortality-related factors in urinary sepsis patients.

## Methods

A retrospective descriptive study of urologic sepsis patients in the ICU from 2008 to 2010. Clinical, epidemiological and outcome variables were analysed. Quantitative variables are expressed as either mean and standard deviation or as median and interquartile range for asymmetric variables. Qualitative variables are expressed as percentages and absolute values. Mann-Whitney's U test and Fisher's exact test were applied (a error was 5% in both cases), as well as binary logistic regression for multivariate analysis.

## Results

There was a total number of 44 patients (aged 59.39 ± 17.71; 63.8% females). APACHE II score upon admission was 18 ± 6. Out of these patients, 27.3% showed no underlying disorder and 18.2% (no = 8) showed chronic renal failure; 25% were immunodepressed patients; 31% underwent urinary instrumentation in the previous 15 days, yet only three of them had undergone permanent urine catheterization. Observed mortality was 25%, while sepsis-related mortality was 22.7%. The patients who died were, on average, older that those who survived (67.9 ±7 10.2 vs. 56.8 ± 18.7; *P *= 0.02). Besides, the former also reported greater delay in turning to the hospital after symptom onset (13.4 ± 6.6 vs. 6.2 ± 4.7 days; *P *= 0.0001). Immunodepressed patients presented higher mortality rate: OR 8.7 (95% CI 1.7 to 42.3), as well as those who underwent inappropriate initial antibiotic treatment: OR 10.8 (95% CI 2.1 to 54.7). No relation was observed between germ typology or resistance to β-lactam antibiotics and mortality. After adjustment of mortality due to APACHE II score upon admission, delay in the onset of appropriate antibiotic treatment was an independent predictor of mortality in our patients: OR 1.2, 95% CI (1.02 to 1.42).

## Conclusion

Urinary sepsis mortality is associated with late-onset and/ or inappropriate antibiotic use, as well as with immunodepression and advanced age.
